# Managing Hospital Capacity: Achievements and Lessons from the COVID-19 Pandemic

**DOI:** 10.1017/S1049023X22001169

**Published:** 2022-10

**Authors:** Enrico R. de Koning, Mark J. Boogers, Saskia LMA Beeres, Iwona D. Kramer, Wouter J. Dannenberg, Martin J. Schalij

**Affiliations:** 1. Regional Capacity and Patient Transfer Service (RCPS) West-Netherlands, Leiden, the Netherlands; 2. Leiden University Medical Center, Leiden, the Netherlands; 3. Leiden University Medical Center Capacity Centre (LUCC), Leiden, the Netherlands; 4. Regional Consultative Body Acute Care; in Dutch: Regionaal Overleg Acute Zorg (ROAZ) West, Leiden, the Netherlands

**Keywords:** capacity, coordination, COVID-19, transfer, triage

## Abstract

**Introduction:**

The coronavirus disease 2019 (COVID-19) pandemic challenged health care systems in an unprecedented way. Due to the enormous amount of hospital ward and intensive care unit (ICU) admissions, regular care came to a standstill, thereby overcrowding ICUs and endangering (regular and COVID-19-related) critical care. Acute care coordination centers were set up to safely manage the influx of COVID-19 patients. Furthermore, treatments requiring ICU surveillance were postponed leading to increased waiting lists.

**Hypothesis:**

A coordination center organizing patient transfers and admissions could reduce overcrowding and optimize in-hospital capacity.

**Methods:**

The acute lack of hospital capacity urged the region West-Netherlands to form a new regional system for patient triage and transfer: the Regional Capacity and Patient Transfer Service (RCPS). By combining hospital capacity data and a new method of triage and transfer, the RCPS was able to effectively select patients for transfer to other hospitals within the region or, in close collaboration with the National Capacity and Patient Transfer Service (LCPS), transfer patients to hospitals in other regions within the Netherlands.

**Results:**

From March 2020 through December 2021 (22 months), the RCPS West-Netherlands was requested to transfer 2,434 COVID-19 patients. After adequate triage, 1,720 patients with a mean age of 62 (SD = 13) years were transferred with the help of the RCPS West-Netherlands. This concerned 1,166 ward patients (68%) and 554 ICU patients (32%). Overcrowded hospitals were relieved by transferring these patients to hospitals with higher capacity.

**Conclusion:**

The health care system in the region West-Netherlands benefitted from the RCPS for both ward and ICU occupation. Due to the coordination by the RCPS, regional ICU occupation never exceeded the maximal ICU capacity, and therefore patients in need for acute direct care could always be admitted at the ICU. The presented method can be useful in reducing the waiting lists caused by the delayed care and for coordination and transfer of patients with new variants or other infectious diseases in the future.

## Introduction

Health care systems are increasingly under pressure. In Western countries, the ageing population leads to a growing demand for health care and hospital capacity.^1^ Paradoxically, the ageing population also results in a decrease in available workforce and thus available health care employees.^2^ This, combined with the aim to lower health care expenditures, drives hospitals in the Netherlands to be run efficiently, or in other words, on the minimum required in terms of bed capacity and available staff. Although the system may be efficient, in times of extreme health care demands, the system comes to a halt.

The coronavirus disease 2019 (COVID-19) pandemic challenged the Dutch health care system in an unprecedented way. During the first wave of infections, from March till May 2020, hospitals came under an immense pressure within a few days. As a first step, emergency departments (EDs) and intensive care units (ICUs) tried to increase the number of available beds to obtain extra surge capacity for COVID-19 patients.^3–5^ However, this meant regular care came to an abrupt standstill, and even critical non-COVID-19 (ICU) care was difficult to manage safely. Still, a large number of patients was in need of invasive respiratory support and thus ICU treatment. The ICUs were overcrowded, and accordingly, interventions after which ICU surveillance is required were postponed.^6,7^ Unfortunately, this pattern repeated during subsequent waves in 2020 and 2021, leading to more postponed care and an increasing waiting list. In April 2022, the Dutch Health Authority (NZa; Utrecht, The Netherlands) estimated that between 100,000-120,000 patients are still on waiting lists for surgical treatment in the Netherlands caused by postponed care during the COVID-19 pandemic.

Before the COVID-19 pandemic, when hospitals had insufficient capacity for patient admissions, patients were transferred between hospitals organized between treating physicians, and the nearest hospital would be the first option for a patient transfer. When this hospital lacked capacity for admission, the physician would contact another regional hospital, and so forth. This system works fine when just one hospital encountered capacity issues. However, when these issues become regional or (inter)national, this system is bound to fail. Clinicians would have to spend an enormous amount of time coordinating patient transfers. Importantly, overcrowding decreases the quality of care, leads to worse patient outcomes, and increases mortality.^8–10^ Furthermore, if ICU capacity is insufficient, patients would need to be triaged for access and, the worst-case scenario, would have to be denied access to life-saving care on the ICU based on, for example, age or previous medical history.^11–14^ This scenario must be avoided at all cost.

It seems that the coronavirus is here to stay, and furthermore, it is likely that in the future, other variants or entirely different infectious diseases may become wide-spread in the current globalized world. These flare-ups of infectious diseases will put direct pressure on hospital capacity. Optimizing capacity management in moments of acute need is of upmost importance, since the main issues driving capacity problems (ageing of the population and health care personnel shortages) cannot be changed nor are they easily rectified. Therefore, to prevent hospitals from overcrowding and maintain (ICU) capacity, a new system to adequately coordinate patient transfers between hospitals was (and is) urgently needed. Not solely for COVID-19 patients during this pandemic, but also for other infectious diseases in the future, and to take care of patients whose treatment was postponed or who failed to visit the ED during the COVID-19 pandemic.^15–17^

During the first wave of the COVID-19 pandemic in the Netherlands, acute care coordination center(s) were set up to manage regional and national hospital capacity for COVID-19 patients. This paper presents the achievements of the COVID-19 Regional Capacity and Patient Transfer Service (RCPS) for the region West-Netherlands. Furthermore, the achievements are put in perspective for current and future stress tests of the health care system.

## Methods

The acute lack of hospital capacity for COVID-19 patients in March 2020 urged physicians, physician-researchers, nurses, data-analysts, general physicians (GPs), nursing homes, and executives to form a new regional system for patient triage and transfer: the RCPS West-Netherlands. The region West-Netherlands is a relatively densely populated urban region comprising seven hospitals servicing approximately 1.5 million people with a regular ICU capacity of 99 beds. The aim of the RCPS West-Netherlands was to ensure that the seven hospitals in the region would share their COVID-19 caseload equally to maximize quality of care for all patients in all hospitals. The presented retrospective observational study assessed hospital capacity and patient transfers throughout the region.

Furthermore, a National Capacity and Patient Transfer Service (LCPS) aimed to equally spread COVID-19 patients among the entire country in corroboration with regional task forces for every region in the Netherlands.^18^ Of importance, all patients in the Netherlands are obliged to have health care insurance from one of the available, competing health care insurers. After the first wave (March-May 2020) of the COVID-19 pandemic, all regions in the Netherlands agreed to meet a “fair share” of COVID-19 patients as defined by the LCPS. This fair share was based on the ratio of regular ICU capacity between all 10 regions in the Netherlands. When then the entire region would be overcrowded, the RCPS West-Netherlands contacted the LCPS for patient transfers to hospitals from other regions. The LCPS would then select the appropriate hospital and further arrange transfers in the same way as indicated above. When other regions in the Netherlands were overcrowded, the RCPS received (and distributed) patients within the region. Adequate patient distribution by the RCPS West-Netherlands was based on two pillars: acute and up-to-date information on hospital capacity and a system for triage and transfer.

### RCPS – Hospital Capacity Data

**Figure 1.**
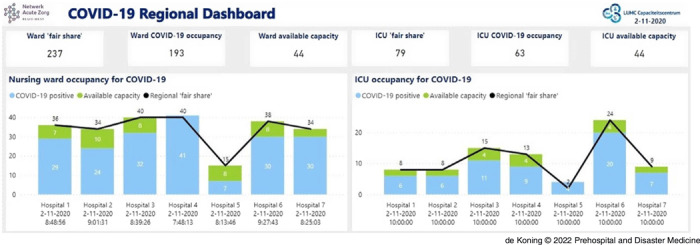
Overview of Available Beds (green) and Occupied Beds (light blue) for Nursing Ward (left panel) and ICU (right panel) for COVID-19. Note: The black line displays the regional “fair share.” Abbreviations: COVID-19, coronavirus disease 2019; ICU, intensive care unit.

For information on hospital capacity, the first step was to provide insight in the number of admitted COVID-19 patients. Initially, automatic aggregating from electronic medical records was not possible as there was no registration standard for COVID-19. Accordingly, each hospital appointed one person to gather data on the number of COVID-19 patients in their hospital, both on the ward and on the ICU. In addition, the number of available ward and ICU beds was registered as well as the ICU occupation of non-COVID-19 patients. These data were automatically uploaded to online spreadsheets developed by the RCPS West-Netherlands, and contact was only needed if data were missing. Based on these data, graphs on COVID-19 occupancy and availability of beds were made. These graphs were openly accessible for all health care professionals in the region on a newly developed platform (Figure 1). After the first COVID-19 wave, from March-May 2020, regional agreements were made on the fair share of COVID-19 patients among the seven regional hospitals. After these agreements were in place, the platform also displayed whether a hospital had sufficient capacity to accept patients from other hospitals (in green) or whether a hospital was over the agreed fair share (black line) and could request transfer of patients. Furthermore, the platform noted the amount of patient movement requests (PMRs) and their current status. Data were requested at fixed times on the day depending on the national and regional risk level (which were developed in June 2020 and changed according to the number of COVID-19 infections and COVID-19 hospital occupancy). The data were updated once on the vigilant level, twice on the worrisome and serious levels, and three times a day on the profoundly serious level.

### RCPS – Patient Selection for Transfer

**Figure 2.**
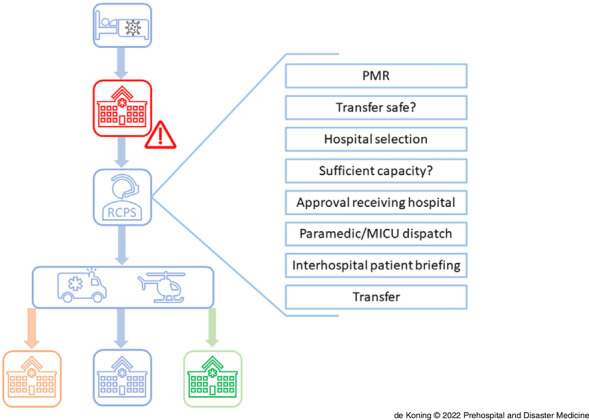
System for Triage of COVID-19 Patients. Note: The overcrowded hospital (red) contacts the RCPS West-Netherlands triage hotline for a PMR. If transfer is safe, the RCPS West-Netherlands selects the best suited hospital for transfer and transfers to one of the available hospitals (orange, blue, green). Abbreviations: COVID-19, coronavirus disease 2019; RCPS, Regional Capacity and Patient Transfer Service; PMR, patient movement request; MICU, Mobile Intensive Care Unit.

The second pillar of the RCPS West-Netherlands was to select patients who were deemed safe for transfer, and subsequently transfer them to the best suited hospital. Physicians, physician-researchers, and nurses were responsible for this triage system (Figure 2) and were continuously available on a central hotline (24/7). When the ED, ward, and/or ICU became overcrowded, a hospital could request patient transfer(s). In the “Patient Movement Request Form,” all information required to decide whether safe transfer was possible was collected, including age, gender, medical history, current medical status, vital parameters, and, if intubated, mode of therapy used in conjunction with mechanical ventilation. In general, transfers were considered safe if the oxygen suppletion, both for nursing ward and ICU patients, was stable for more than two hours. If the triagist considered transfer safe, the receiving hospital was selected based on the patients’ clinical parameters and/or history. Patients potentially requiring Extra Corporeal Membrane Oxygenation were transferred to an intervention center with these capabilities. Furthermore, patients with prior organ transplants were preferably transferred to a transplant center. Subsequently, capacity was checked in the concerned hospital (as illustrated in Figure 1), whereafter the attending physician from the receiving hospital was contacted. After final approval of this physician, transport was organized by either paramedics (for ward patients) or by Mobile Intensive Care Units (MICU) for ICU patients. The Emergency Medical Services control room was contacted, determined regional ambulance availability, and chose a suitable and available ambulance. Patients would be transported by the nurse paramedics from the region of the receiving hospital. The physician who requested the transfer was contacted to announce the estimated time of pick-up. The patient’s current attending physician would be obligated to contact the physician to which authority the patient would be transferred for a full and up-to-date patient briefing. Furthermore, a printed medical history was brought with the nurse paramedic for the receiving hospital. Physicians could always contact each other themselves or through the RCPS if there were any questions after patient transfer. After this final confirmation, transportation was performed.

Once the RCPS West-Netherlands platform has been developed using both the information on current capacity for COVID-19 patients as well as the system for adequate triage and safe transfer, it is easily activated or inactivated. When COVID-19 infections are scarce, the system is dormant, and when necessary, woken up within a day.

## Results

From March 2020 through December 2021 (22 months), the RCPS West-Netherlands was requested to transfer 2,434 COVID-19 patients. Of the 2,434 patients, 714 (29%) were not transferred, either because transfer was considered unsafe (n = 180; 25%) or because the transfer request was cancelled (n = 534; 75%).

**Figure 3.**
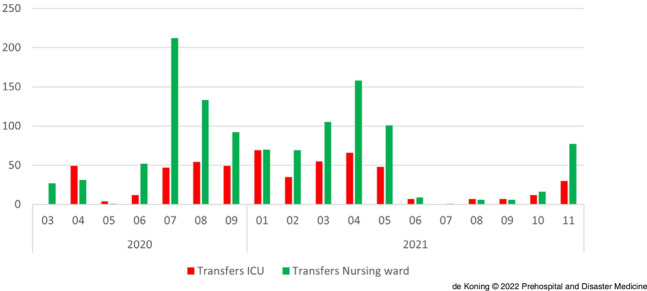
Number of Transfers by RCPS West-Netherlands for ICU (red) and Nursing Ward (green) per Month from March 2020 - November 2021. Abbreviations: RCPS, Regional Capacity and Patient Transfer Service; ICU, intensive care unit.

Eventually, 1,720 patients with a mean age of 62 (SD = 13) years were transferred with the help of the RCPS West-Netherlands. This concerned 1,166 ward patients (68%) and 554 ICU patients (32%). Figure 3 shows the number of transfers arranged by the RCPS West-Netherlands for nursing ward and ICU patients per month. Of interest, 830 (48%) patients were transferred from a hospital within the region to another hospital within the region, 733 (43%) patients were transferred from a hospital within the region to a hospital outside the region, whereas 157 patients (9%) were transferred from another region to this region in collaboration with the LCPS.

### RCPS Transfers and ICU/Ward Occupation on a Regional Level

The combined West-Netherlands regional COVID-19 hospital occupation for nursing wards and ICUs is displayed in Figure 4a and Figure 4b. Figure 4a illustrates that the nursing ward occupation (blue) benefitted from RCPS transfers to hospitals outside the region in October-November 2020 and April-May 2021 (as illustrated by the orange highlights). Figure 4b reveals the same phenomenon over time for ICU occupation. Importantly, it also shows that after the first wave, the regional total ICU occupation never exceeded the regional regular (pre-COVID-19) ICU capacity of 99 beds (displayed in red). Accordingly, the RCPS-arranged transfers helped to maintain ICU capacity for non-COVID-19 patients.

**Figure 4a.**
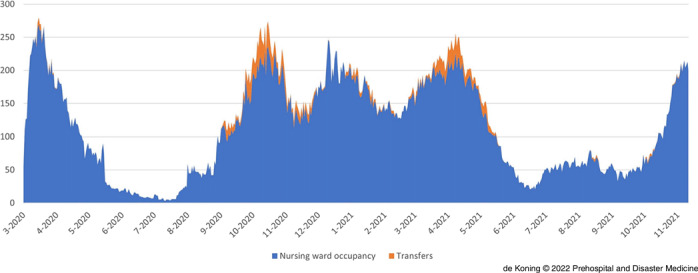
Nursing Ward Occupancy for COVID-19 (blue) in West-Netherlands and the Potential Occupancy if there had been No Patient Transfers (orange). Abbreviation: COVID-19, coronavirus disease 2019.

**Figure 4b.**
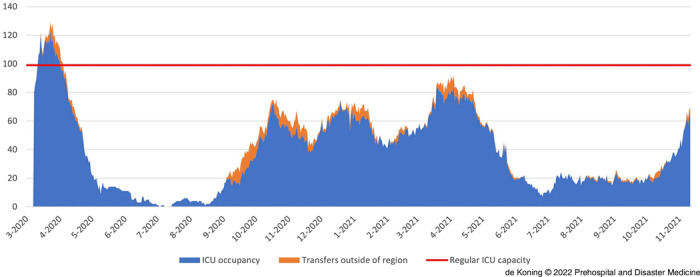
ICU Occupancy for COVID-19 (blue) in West-Netherlands and the Potential Occupancy (orange). Note: The red line corresponds with the regular ICU capacity for all patients. Abbreviations: COVID-19, coronavirus disease 2019; ICU, intensive care unit.

### RCPS Transfers and ICU Occupation on a Hospital Level

Figure 5a and Figure 5b give more insight into the effect of the RCPS-arranged transfers on the ICU occupation on a single hospital level. The hospital of which these data are displayed in Figure 5a is situated in a highly urban area. The percentage of vaccinated patients in this area was relatively low as compared to the rest of the region. The number of patients from this area far exceeded the available capacity. Pre-COVID-19, this hospital had an average operational ICU capacity of 20 beds. Figure 5a shows the true COVID-19 ICU occupation in blue and the fictitious extra ICU occupation in orange if patients hadn’t been transferred. This fictious occupancy was calculated by multiplying the transfers from this hospital to any other hospital with the average ICU stay for COVID-19 patients (11 days); means were compared with an unpaired t-test. From October 2020 through March 2021, the mean (standard deviation) true ICU occupancy for COVID-19 patients for this hospital was 8.5 (SD = 2.6) beds. However, if patients hadn’t been transferred, the potential ICU occupation would have been 12.8 (SD = 4.4) beds (P <.001).

**Figure 5a.**
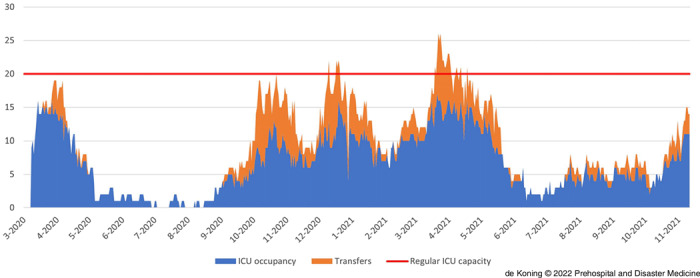
True ICU Occupancy of COVID-19 Patients of a Single Hospital in a Highly Urban Area (blue) and Potential ICU Occupancy if Patients hadn’t been Transferred (orange). Abbreviations: COVID-19, coronavirus disease 2019; ICU, intensive care unit.

**Figure 5b.**
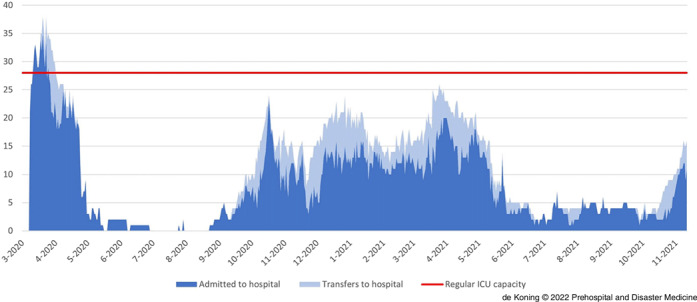
Total ICU Occupancy of a Tertiary Care Hospital Consisting of Patients Directly Admitted to the Hospital (dark blue) and Patients Transferred to the Hospital (light blue). Abbreviation: ICU, intensive care unit.

The hospital of which the data are displayed in Figure 5b is a tertiary care hospital with advanced intervention capabilities. This hospital has a large reserve capacity because of the large amount of semi-elective interventions that could be postponed. Furthermore, due to the fact this concerns a teaching hospital, teachers and students could be mobilized to help with patient care and logistics. Pre-COVID-19, the hospital had an average operational ICU capacity of 28 beds. Patients directly admitted to this hospital are shown in dark blue and patients transferred to this hospital are shown in light blue. As illustrated, the COVID-19 ICU occupation in this hospital is particularly characterized by the RCPS-arranged transfers towards this hospital. From October 2020 through March 2021, the mean true ICU occupancy for COVID-19 patients for this hospital was 15.1 (SD = 4.6) beds. However, if patients hadn’t been transferred, the fictitious ICU occupation would have been 10.5 (SD = 3.9) beds (P <.001).

### RCPS Transfers and ICU Occupation on a National Level

**Figure 6.**
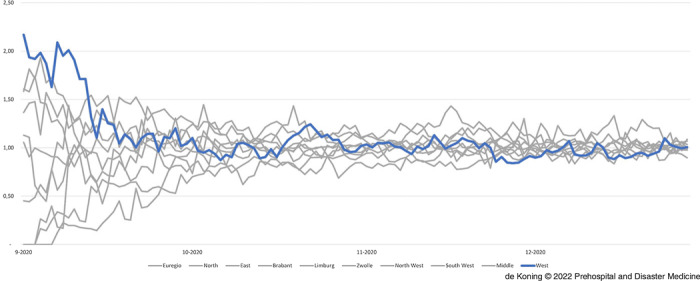
National ICU Occupancy Relative to “Fair Share” for the Second Wave Starting in September 2020. Note: West-Netherlands in blue, all other regions in the Netherlands in grey. Abbreviation: ICU, intensive care unit.

From September 2020 onwards, the national agreements on fair share were established and put in practice through collaboration with the LCPS. Figure 6 displays the ICU occupancy for COVID-19 patients relative to the agreed fair share from September 2020 through February 2021. Initially, the ICU occupancy of hospitals in the region West-Netherlands was more than 2.0-times higher than the agreed fair share, as seen at the start of Figure 6 on the blue line. Transfers from COVID-19 patients to other regions in the Netherlands, a direct effect of close collaboration between the RCPS West-Netherlands and the LCPS, decreased the relative ICU occupancy. As seen in Figure 6, this led to an equal spread of COVID-19 patients throughout the Netherlands.

## Discussion

The current study concludes that the health care system benefitted from the RCPS as both ward and ICU occupation were evenly distributed in the region West-Netherlands. After establishing the RCPS, ICU occupation never exceeded the maximal ICU capacity, and accordingly, ICU capacity remained for patients in need of urgent care. By collaborating with the LCPS, the RCPS contributed to an even distribution (or “fair share”) of COVID-19 patients among the country. In future health care capacity stress tests, such as flare-ups of infectious diseases or increasing waiting lists, the RCPS can easily and rapidly be implemented again.

World-wide, the COVID-19 pandemic challenged health care systems, as huge amounts of patients required hospitalization while, at the same time, health care workers dropped out due to illness or quarantine. As a consequence, regular care came to a standstill, acute care was critically endangered, and surgeries after which ICU surveillance is required were postponed. To stop this and prevent recurrence during new flare-ups, building in flexibility in the organization of health care is mandatory. Naturally, various approaches are imaginable.

The most intuitive approach, and first step, for a hospital is to increase the number of available beds for COVID-19 patients, both on ICUs as well as on wards. This can be achieved by using operating rooms for COVID-19 care,^19^ building alternate care sites,^20^ or quickly training nurses to care for COVID-19 patients.^21^ Dutch hospitals are typically run with minimal capacity and personnel in order to minimize health care expenditures, which is illustrated by a relatively low number of available ICU beds (6.7 per 100,000 inhabitants) as compared to other countries, such as Germany (33.4 ICU beds per 100,000 inhabitants).^22^ Accordingly, the abilities to increase the number of beds within a hospital in the short and medium term were limited.

Another option to increase the amount of available beds is to discharge patients as early as possible, thereby freeing up capacity for newly admitted patients. E-health applications allowed patients to be monitored at home, which also helps to reduce the demand of hospital capacity.^23^ The GPs and nursing homes in the region West-Netherlands participated in the consultative bodies to set up guidelines for early discharge of patients. Nursing homes even freed up beds especially designed for COVID-19 patients ready for discharge from the hospital but not ready for discharge home. The next step is critical assessment of which patients benefit most from hospital admission. These assessments should preferably be made in the prehospital setting, before patients are admitted to the ED. Ambulance nurses and GPs can perform this prehospital triage and leave patients at home when ED referral is not necessary, as was done for cardiac complaints in the HART-c study.^24^

When increasing the number of beds and improving prehospital triage offered insufficient solace, collaboration with neighboring hospitals is a next step. The Modena Taskforce response in Italy implemented a dashboard called Pagoda to show the number of positive tests and ICU occupancy throughout the region. Furthermore, the Modena Taskforce took an active role in reallocation of resources to hospitals and in the community.^25^ A dashboard was also developed in Picardy, France to coordinate and facilitate the admission of critically ill COVID-19 infected patients. An anesthesiologist, who also functions as an intensivist, was on call for EDs and ICUs of the region. The dashboard showed the occupancy of all regional ICUs and the dispatcher could therefore select the most appropriate hospital for all announced COVID-19 patients.^26^ In the Netherlands, regional and national coordination networks were set up; the early experiences of the Acute Care Organization in the Amsterdam region have been published earlier.^18^ The method of coordination and transfer in the early experiences in Amsterdam was similar to the method reported in the current study. However, the current study used a newly developed dashboard to gather insight in data of importance for COVID-19 capacity, such as the amount of positive tests, as well as nursing ward and ICU occupancy throughout the entire region. If regional collaboration would not suffice, physicians would have to triage and deny patients access to wards or ICU based on ethical reasons. This worst-case scenario, “code black,” is to be avoided at all costs, although these scenarios were trained in all hospitals within the region.

The RCPS combined information on capacity and triage capabilities to effectively transfer patients throughout the region and the country. Just as the Picardy study, a dispatcher was on call for the ICU in the entire region. However, of added value, the RCPS also arranged (safer and easier) transfers for nursing ward COVID-19 patients. The method of coordination and transfer in the currently presented study was similar to the method reported in the early experiences in Amsterdam and the national network. However, they only presented the experiences of the first two months of coordinating transfers. The current study shows the results from 22 months of coordination during differing numbers of COVID-19 infections. Due to the transfers from the RCPS, the nursing ward and ICU occupancy was lower and vital capacity was maintained for non-COVID-19 patients. Most importantly, the ICU occupancy for the entire region never exceeded the maximal capacity like it did in the first wave. Hospitals with high influx of COVID-19 patients were able to transfer patients to other hospitals in the region who had a higher reserve capacity or a lower influx of patients. The transfers aided in significantly reducing the ICU occupancy of overcrowded hospitals.

## Limitations

Several limitations should be taken into account when interpreting the results. At first, the fictitious ICU occupancy was based on the assumption that an ICU admission would have lasted 11 days if patients would not have been transferred. Therefore, the “true” ICU occupancy could have been lower or higher than the “aggregated” ICU occupancy shown here. Furthermore, it remains to be proven that this system could be replicated in all other countries; however, when data dashboards are available (such as those in Modena and Picardy), dispatch and transferring patients as was reported here is easily implementable. Moreover, the information on capacity requires cooperation between different, sometimes financially competitive, hospitals, which was achieved during this time of crisis. However, it is to be seen whether this system holds up when the crises are less acute.

The currently presented study proves that cooperation between hospitals increases the available capacity through improved use of existing health care resources, thereby preventing critical overcrowding of wards and ICUs. As a further lesson, the RCPS showed that transparency, in this case by making a dashboard available with insight in all COVID-19-related admissions and infections, leads to increased willingness for cooperation for all relevant health care professionals throughout the region and the country.

Although the Omicron variant of SARS-CoV-2 might be less fatal and lead to less hospitalizations, new waves or variants of COVID-19, or indeed other infectious diseases, might cause high numbers of infections and hospitalizations in the future. Also, medical interventions and treatments were postponed to provide capacity for COVID-19 patients on the nursing wards and ICUs. This has led to enormous waiting lists throughout the country for non-COVID-19-related care. Since the presented method of patient transfer and distribution is easily implemented after all logistics are in place, the RCPS and LCPS could potentially aid in eliminating the waiting lists (equally) throughout the country. The data on COVID-19 care could be replaced with information on capacity of postponed interventions, such as operating room, nurse, and surgeon availability, from all hospitals in the Netherlands. The COVID-19 (hospital capacity) crisis has shown that there is strength in cooperation and full transparency of available health care resources.

## Conclusion

The health care system in the region West-Netherlands benefitted from the RCPS for both ward and ICU occupation. Due to the coordination by the RCPS, regional ICU occupation never exceeded the maximal ICU capacity, and therefore, patients in need for acute direct care could always be admitted at the ICU. The presented method can be useful in reducing the waiting lists caused by the delayed care and for coordination and transfer of patients with new variants or other infectious diseases in the future.
